# Non-Invasive Assessment of Locally Overexpressed Human Adenosine 2A Receptors in the Heart of Transgenic Mice

**DOI:** 10.3390/ijms23031025

**Published:** 2022-01-18

**Authors:** Daniel Gündel, Thu Hang Lai, Sladjana Dukic-Stefanovic, Rodrigo Teodoro, Winnie Deuther-Conrad, Magali Toussaint, Klaus Kopka, Rareş-Petru Moldovan, Peter Boknik, Britt Hofmann, Ulrich Gergs, Joachim Neumann, Peter Brust

**Affiliations:** 1 Department of Neuroradiopharmaceuticals, Institute of Radiopharmaceutical Cancer Research, Research Site Leipzig, Helmholtz-Zentrum Dresden-Rossendorf, 04813 Leipzig, Germany; t.lai@hzdr.de (T.H.L.); s.dukic-stefanovic@hzdr.de (S.D.-S.); r.teodoro@hzdr.de (R.T.); w.deuther-conrad@hzdr.de (W.D.-C.); m.toussaint@hzdr.de (M.T.); k.kopka@hzdr.de (K.K.); r.moldovan@hzdr.de (R.-P.M.); p.brust@hzdr.de (P.B.); 2 Department of Research and Development, ROTOP Pharmaka Ltd., 01328 Dresden, Germany; 3 Faculty of Chemistry and Food Chemistry, School of Science, Technical University Dresden, 01069 Dresden, Germany; 4 Institute for Pharmacology and Toxicology, University of Muenster, 48149 Muenster, Germany; boknik@uni-muenster.de; 5 Cardiac Surgery, Medical Faculty, Martin Luther University of Halle-Wittenberg, 06120 Halle, Germany; britt.hofmann@uk-halle.de; 6 Institute for Pharmacology and Toxicology, Martin Luther University of Halle-Wittenberg, 06112 Halle, Germany; Ulrich.gergs@medizin.uni-halle.de (U.G.); joachim.neumann@medizin.uni-halle.de (J.N.); 7 The Lübeck Institute of Experimental Dermatology, University Medical Center Schleswig-Holstein, 23562 Lübeck, Germany

**Keywords:** [^18^F]FLUDA, A_2A_ adenosine receptor, PET, myocardium, heart failure

## Abstract

A_2A_ adenosine receptors (A_2A_-AR) have a cardio-protective function upon ischemia and reperfusion, but on the other hand, their stimulation could lead to arrhythmias. Our aim was to investigate the potential use of the PET radiotracer [^18^F]FLUDA to non-invasively determine the A_2A_-AR availability for diagnosis of the A_2A_R status. Therefore, we compared mice with cardiomyocyte-specific overexpression of the human A_2A_-AR (A_2A_-AR TG) with the respective wild type (WT). We determined: (1) the functional impact of the selective A_2A_R ligand FLUDA on the contractile function of atrial mouse samples, (2) the binding parameters (*B*_max and_ *K*_D_) of [^18^F]FLUDA on mouse and human atrial tissue samples by autoradiographic studies, and (3) investigated the in vivo uptake of the radiotracer by dynamic PET imaging in A_2A_-AR TG and WT. After A_2A_-AR stimulation by the A_2A_-AR agonist CGS 21680 in isolated atrial preparations, antagonistic effects of FLUDA were found in A_2A_-AR-TG animals but not in WT. Radiolabelled [^18^F]FLUDA exhibited a *K*_D_ of 5.9 ± 1.6 nM and a *B*_max_ of 455 ± 78 fmol/mg protein in cardiac samples of A_2A_-AR TG, whereas in WT, as well as in human atrial preparations, only low specific binding was found. Dynamic PET studies revealed a significantly higher initial uptake of [^18^F]FLUDA into the myocardium of A_2A_-AR TG compared to WT. The hA_2A_-AR-specific binding of [^18^F]FLUDA in vivo was verified by pre-administration of the highly affine A_2A_AR-specific antagonist istradefylline. Conclusion: [^18^F]FLUDA is a promising PET probe for the non-invasive assessment of the A_2A_-AR as a marker for pathologies linked to an increased A_2A_-AR density in the heart, as shown in patients with heart failure.

## 1. Introduction

Multiple effects of adenosine in humans and animals have been described for many years. In the heart, adenosine, potentially released from myocardial ATP, reduces the heart rate and dilates coronary arteries [1]. It elicits negative chronotropic (sinus node), negative dromotropic (AV-node), and negative inotropic (atrial tissue) effects in the hearts of mice and humans [1]. In the ventricle of most mammalian species, including humans, adenosine diminishes force only in the presence of cAMP-increasing agents, like β-adrenoceptor agonists or phosphodiesterase inhibitors. Adenosine binds to different adenosine receptors, which are classified into different subtypes (A_1_-AR, A_2A_-AR, A_2B_-AR, and A_3_-AR) [1,2]. They are located in the outer cell membrane of cardiomyocytes, endothelial cells, fibroblasts, erythrocytes, leucocytes, and smooth muscle cells and coupled to GTP-binding proteins to trigger intracellular signalling pathways. Thereby, the binding of adenosine to the high-affinity A_1_-AR and A_2A_-AR regulates the intracellular cAMP content by opposing effects on the adenylyl cyclase activity (A_1_-AR: inhibition, A_2A_-AR: activation) [3,4]. Additionally, inotropic effects elicited by β-adrenergic stimulation (cAMP elevation) are antagonised by the A_1_-AR adenosine signalling in cardiomyocytes in the atrium and the ventricle [5,6]. The adenosine signalling by A_2A_-AR not only attenuates the A_1_-AR signalling, but also leads to a dilatation of the cardiac vasculature [7] and could indirectly increase the contractility by increasing the oxygen supply [8]. Thus, the cardioprotective effects of adenosine may involve signalling via A_1_-AR and A_2A_-AR [9].

Elevated A_2A_-AR expression was found in the atrium of patients with atrial fibrillation [10,11], while it was decreased in ventricular tissue of patients with chronic heart failure [12]. The beneficial effects of A_2A_AR agonists, such as LASSBio-294, prevented cardiac dysfunction in a rat model [13]. Similar effects were found for the A2AAR agonists ATL-193 and ATL-146e in postischemic stunning of the myocardium in a canine model [14].

Obviously, the protective but also deleterious effects of adenosine depend not only on the plasma and interstitial concentrations of adenosine, but also on the expression and receptor density on cell membranes [15]. Therefore, non-invasive imaging of the cardiac A_2A_R availability is a promising, non-invasive tool to stratify prognosis of cardiac damage, but also for the determination of the receptor occupancy by potential A_2A_-AR agonists.

**Figure 1:**
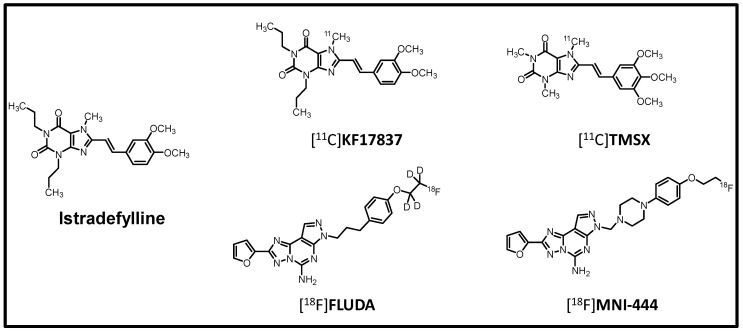
Chemical structures of A_2A_-AR PET radioligands and the A_2A_-AR antagonist istradefylline used in the present study.

A_2A_-AR imaging has successfully been performed in neurological disorders using positron emission tomography (PET) with [^11^C]preladenant and, recently, [^18^F]MNI-444 in a clinical phase III study [16,17]. The *K*_i_ of [^18^F]MNI-444 towards human A_2A_-AR is comparably high (2.8 nM) [17]. Hence, a striatum-to-cerebellum ratio (measure of specific binding) of about 12 was found at 60–70 min after injection in humans [17]. Our recently developed A_2A_-AR-specific PET radiotracer [^18^F]FLUDA (human A_2A_-AR: *K*_i_ = 0.6 nM; human A_1_-AR: *K*_i_ = 767 nM) appears highly suitable for the non-invasive A_2A_-AR imaging of mice and piglet brains [18]. In terms of assessing the A_2A_-AR availability in the heart, aiming at the visualisation of pathologically relevant changes, earlier attempts were performed by Ishiwata and colleagues using the A_2A_-AR radioligands [^11^C]KF17837 and [^11^C]TMSX (Figure 1) in rabbits and humans, whereat [^11^C]TMSX revealed good properties for PET imaging and high plasma stability [19,20].

With that regard, the aim of this study was to evaluate the potential of [^18^F]FLUDA for A_2A_-AR imaging by PET in a mouse model with a functional myocardial overexpression of the human A_2A_-AR [21].

## 2. Results

### 2.1. Impact of FLUDA on the Atrial Force of Contraction (FOC) in Electrically Stimulated Atrial Preparations

**Figure 2:**
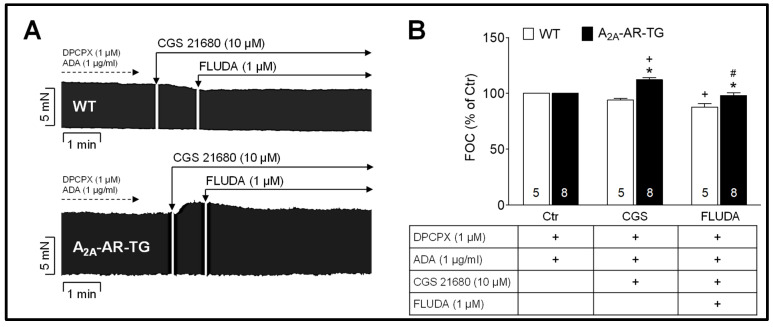
FLUDA (1 µM) inhibits the A_2A_-AR-dependent force of contraction (FOC) in isolated electrically driven left atria from A_2A_-AR transgenic mice (A_2A_-AR-TG) but not from wild-type mice (WT). (**A**) Exemplary original recordings of WT and A_2A_-AR-TG left atria. Control conditions (Ctr) are the inhibition of A_1_-adenosine receptors by 1 µM DPCPX and the degradation of extracellular adenosine by 1 µg/mL adenosine deaminase (ADA). Induction of atrial contraction was achieved by the A_2A_-AR agonist CGS 21680 (10 µM). (**B**) Quantification of left atrial force. Data are means ± SEM; numbers in columns are numbers of atrial preparations and + marks the added substance; * *p* < 0.05 vs. WT; ^+^ *p* < 0.05 vs. Ctr; # *p* < 0.05 vs. CGS (ANOVA).

The yet unknown agonistic or antagonistic action of FLUDA towards the human A_2A_-AR was investigated in electrically stimulated atrial preparations of wild-type mice (WT) and hA_2A_-AR TG (Figure 2). As shown in Figure 2B, the FOC of WT was not changed after adding 10 µM of the A_2A_-AR agonist CGS 21680 (positive inotropic effect) to the organ bath. An anti-inotropic effect towards the human A_2A_-AR was observed after the subsequent adding of 1 µM FLUDA. The FOC was decreased by 15.0 ± 2.4% in the A_2A_-AR TG atria. Hence, these results revealed an antagonistic effect of FLUDA towards a stimulated A_2A_-AR.

### 2.2. In Vitro Binding of [^18^F]FLUDA to the A_2A_-AR in Heart Samples

**Figure 3:**
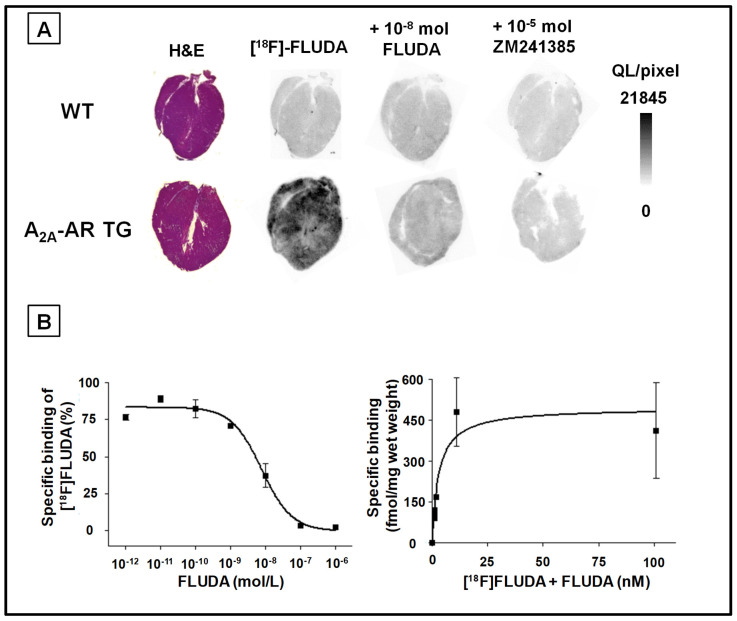
Representative in vitro autoradiographic images of the binding pattern of [^18^F]FLUDA mouse heart slices. (**A**) Hematoxylin/eosin staining (H & E), total binding of [^18^F]FLUDA, homologous (FLUDA), and non-homologous (ZM 241385) displacement of [^18^F]FLUDA; (**B**) representative homologous competition curve of [18F]FLUDA and the saturation curve transformed from competition curve from A_2A_-AR TG hearts. *K*_D_ and *B*_max_ were calculated from the homologous competition of [^18^F]FLUDA with FLUDA by the Cheng-Prusoff equation.

We performed competition assays to determine the A_2A_-AR specificity of [^18^F]FLUDA towards the heart tissue of WT and A_2A_-AR TG (Figure 3). In cardiac cryosections of WT, non-homologous competition with ZM 241385 revealed a specific A_2A_-AR binding of [^18^F]FLUDA of 24.6 ± 9.6% (Figure 3A). However, the low signal of total binding, as well as the homologous competition with FLUDA, suggests a very low A_2A_-AR density, preventing the determination of the endogenous A_2A_-AR receptor density *B*_max_. In cardiac cryosections of A_2A_-AR TG, non-homologous competition with ZM241385 revealed a specific binding of 69.0 ± 6.6% (Figure 3A). A *B*_max_ of 455 ± 78 fmol/g wet weight and a *K*_D_ of 5.9 ± 1.6 nM was determined (Figure 3B). Notably, the specific binding of [^18^F]FLUDA in muscle and lung tissues were comparable in WT and A_2A_-AR TG (Figure S1).

### 2.3. In Vitro Binding Studies of [^18^F]FLUDA in Human Atrial Samples

**Figure 4:**
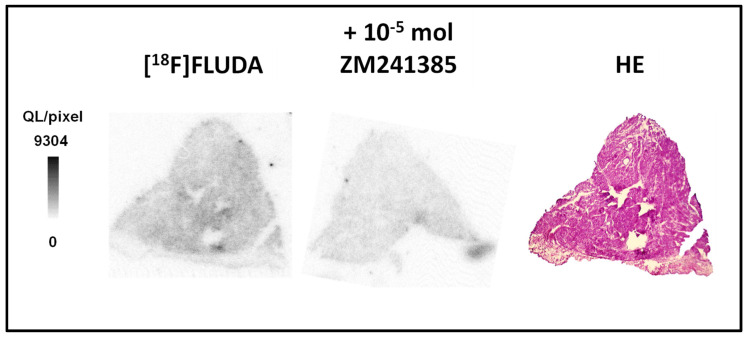
Representative in vitro autoradiographic images of [^18^F]FLUDA binding to human atrial samples, showing the total binding and displacement of the radioligand by the A_2A_-AR-specific receptor antagonist ZM241385, as well as the corresponding HE staining of an atrial cryosection of a patient without heart failure.

In a preliminary study, we used cryosections of human atrial samples of subjects without any diagnosed heart failure for autoradiography with [^18^F]FLUDA and determined an A_2A_-AR-specific binding of 36.3 ± 5.3% (*n* = 2) (Figure 4), which is comparable to the values determined in the WT group of mice, representing a low A_2A_-AR availability in the healthy heart tissue.

### 2.4. In Vivo Uptake into the Heart and Other Tissues of [^18^F]FLUDA in WT and A_2A_-AR TG under Baseline Conditions and after Blocking with Tozadenant and Istradefylline

**Figure 5:**
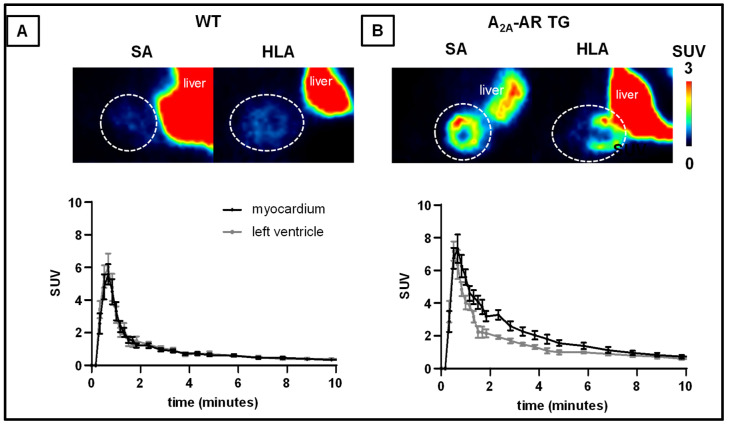
In vivo evaluation of the [^18^F]FLUDA biodistribution in the abdominal heart region by PET imaging. Images show the cardiac planes averaged from 1 to 10 min p.i. in the short axis (SA) and horizontal long axis (HLA) of averaged time frame between three and ten minutes after administration of [^18^F]FLUDA in (**A**) WT and (**B**) A_2A_-AR TG. The heart region is marked with a dotted circle. Mice were pre-treated with vehicle 10 min prior to radiotracer application. Lookup-table decodes for the mean activity concentration given in standardised uptake values (SUV).

Based on the promising in vitro observations, we performed PET studies over 60 min under baseline and blocking conditions to determine the biodistribution of [^18^F]FLUDA (Figure 5 and Figures S2–S5).

**Figure 6:**
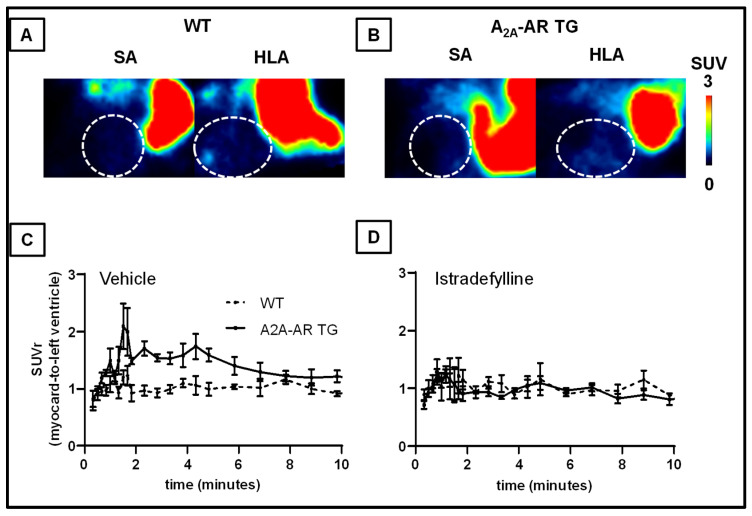
[^18^F]FLUDA time-activity curves (TACs) in the myocardium and the left ventricle (blood compartment). Values are represented as mean standardised uptake values (SUV ± S.E.M.) in the initial 10 min after radiotracer administration in (**A**) WT and (**B**) A_2A_-AR TG (*n* =). (**C**) TACs of the myocardium normalised to the blood compartment, and (**D**) to the muscle (SUVr ± S.E.M.); *n* = 6–8.

Under baseline conditions, we observed an increased initial uptake (1 to 10 min p.i.) of [^18^F]FLUDA in the myocardium of the A_2A_-AR TG compared to WT (Figure 5). The analysis of the time-activity curves (TACs) (Figure 5, Table 1) revealed an earlier time-to-peak value in the blood compartment (−0.2 min, *p* = 0.011) and a higher TAC peak value in the myocardium of A_2A_-AR TG (+10.4%) compared to WT. Hence, the integrated activity concentration over time was higher in both compartments. In the initial phase, a 2.3 times higher AUC value (AUC_1–10_, *p* < 0.001) was found in the myocardium, while it was 1.6 times higher (AUC_1–10_, *p* = 0.004) in the blood, as a result of the functional overexpression of the hA_2A_-AR. To normalise for unspecific physiological effects, AUC_1–10_ ratios (SUVr) of the myocardium to blood (Figure 6C, Table 3) and myocardium to muscle (Table 3) were calculated. They confirmed the increased uptake of [^18^F]FLUDA into the myocardium of A_2A_-AR TG compared to the WT under baseline conditions. Other tissues showed comparable SUVrs in WT and A_2A_AR TG under baseline conditions, suggesting a negligible impact of the functional hA_2A_-AR overexpression in the myocardium on the pharmacokinetics of the radiotracer.

For blocking studies, the A_2A_-AR-specific inhibitor istradefylline (Figure 1) was injected 5 min before the radiotracer to prove the A_2A_-AR-specific uptake of [^18^F]FLUDA into the myocardium (Figure 6, Table 2 and Table 3). In WT, the time to peak in the blood compartment of the left ventricle was earlier compared to the baseline conditions (*p* = 0.017), whereas the TAC peak value in the muscle was slightly increased (SUV of 0.5 ± 0.1 vs. 0.8 ± 0.1, *p* = 0.027) compared to baseline WT. However, we found no significant changes in the AUC_1–10_ and AUC_0–60_ for these and other investigated tissues in comparison to the untreated WT. In A_2A_-AR TG, pre-injection of istradefylline abolished the increased uptake of [^18^F]FLUDA into the myocardium, as shown by the significant reduction of the AUC_1–10_, about 0.6 times (*p* = 0.032 vs. WT). These results were validated by the normalisation of the AUC_1–10_ of the tissues to the blood compartment, as well as to muscle. It was apparent that tissue normalisation to the blood compartment was less prone to fluctuation compared to the muscle, as shown by the SEM values (Table 3); thus, the blood compartment seems more suitable as a reference tissue. Additionally, in the more sensitive ex vivo autoradiography studies, the increased uptake of [^18^F]FLUDA in the murine hearts of A_2A_-AR TG was found even fifteen minutes post-injection and was blocked by pre-administration of istradefylline (Figure S3).

Hence, the high specific binding of [^18^F]FLUDA towards the overexpressed hA_2A_-AR in the myocardium of mice, as shown by the in vitro autoradiography studies, could be validated in vivo by PET imaging.

## 3. Discussion

The present work demonstrates the usefulness of the new radiotracer [^18^F]FLUDA for specific A_2A_-AR imaging by PET in cardiac tissue. We present evidence that (1) FLUDA is a functional antagonist towards the human A_2A_-AR and (2) [^18^F]FLUDA binds specifically and with high binding affinity to the human A_2A_-AR in a mouse model with transgenic overexpression of the receptor in the cardiac tissue and also to A_2A_-AR in human cardiac tissue in vitro. Furthermore, we found an increased uptake in the initial phase of the in vivo biodistribution of [^18^F]FLUDA in the A_2A_-AR overexpressing mouse model by dynamic PET imaging.

Ishiwata and colleagues provided evidence of the successful non-invasive assessment of A_2A_-AR in a human subject by PET imaging using the methylxanthine derivative of KF17837 [^11^C]TMSX [20]. [^11^C]TMSX was later used in subsequent studies to compare the A_2A_-AR density in hearts between endurance athletes and untrained men [22,23]. The non-xanthine derivative [^18^F]FESCH was one of the first ^18^F-labelled A_2A_-AR PET-imaging probes [24,25], and its deuterated isotopologue [^18^F]FLUDA was recently developed and evaluated in vitro as well as in vivo by our group [18]. In the present study, we could show a reduction of the FOC in atrial preparations (stimulated with CGS 21680) from transgenic, but not from wild type mice, after adding FLUDA (Figure 2). Hence, we could confirm the antagonistic action of FLUDA towards the A_2A_-AR by a functional assay.

In a former study with [^18^F]FLUDA, we determined a *K*_D_ value of 4.30 ± 0.73 nM and a *B*_max_ value of 556 ± 143 fmol/mg wet weight in the striatum of healthy CD-1 mice and a *K*_D_ value of 0.68 nM, as well as a *B*_max_ value of 218 fmol/mg wet weight in the striatum of piglets [18], a brain region with a high A_2A_-AR density. In the human heart, the A_2A_-AR is localised at the level of the Z-line of atrial myocytes, where it is co-expressed with α-actinin and the ryanodine receptor [26]. In the present study, we determined comparable A_2A_-AR binding kinetics of [^18^F]FLUDA in cardiac cryosections of A_2A_-AR TG (*K*_D_ of 5.9 ± 1.6 nM and a *B*_max_ of 455 ± 78 fmol/mg protein) as in the striatum of healthy male CD-1 mice, although reliable binding kinetic parameters for cardiac cryosections of female FVB/N mice (WT) could not be determined (Figure 3). We assume that the A_2A_-AR density in the cardiac tissue of WT is low and at the edge of the detection limit of [^18^F]FLUDA, confirming the findings of the functional assays. Binding kinetic studies from other groups with [^3^H]ZM241385 revealed a higher A_2A_-AR density in human heart membrane preparations from patients with chronic heart failure (NYHA functional class III and IV, *B*_max_ = 210 ± 8 fmol/mg protein), which was accompanied with a decreased ligand binding affinity (*K*_D_ = 2.4 ± 0.1 nmol/L) when compared to control (*B*_max_ = 135 ± 5 fmol/mg protein, *K*_D_ = 0.9 ± 0.0 nmol/L) [27]. As an initial step in the present study, we determined a low A_2A_-AR-specific binding with [^18^F]FLUDA in the human atrial samples of patients with no diagnosed heart failure (Figure 4), which is in accordance with the finding in binding studies in cardiac cryosections of WT mice in this study. Hence, we would also expect a good signal-to-background ratio in humans with an increased A_2A_-AR receptor density in the heart. A direct comparison to heart samples of patients with heart failure and atrial fibrillation should be performed in future studies.

A_2A_-AR agonists are often used in the clinic to dilate the coronary arteries in patients and to assess the severity and functional consequences of impaired vasodilation in angina pectoris [28]. However, [^18^F]FLUDA is not expected to be useful to image the coronary A_2A_-AR density, specifically, as the amount of smooth muscle cells and endothelial cells is much lower compared to the number and volume of cardiomyocytes. Thus, we would expect a binding of [^18^F]FLUDA mainly to the A_2A_-AR in cardiomyocytes. The in vivo studies in mice revealed increased uptake of [^18^F]FLUDA under baseline conditions into the myocardium of A_2A_-AR TG compared to WT. However, this was restricted to the initial phase after administration of the radioligand. In a single ex vivo autoradiography, we could confirm increased A_2A_-AR-specific uptake into the myocardia of A_2A_-AR TG even after 15 min p.i. (Figure S3). An accumulation over time of [^18^F]FLUDA into the cardiac tissue of mice could not be observed as it was shown in other studies for [^11^C]TMSX [20]. Interestingly, in that study, the cardiac uptake of [^11^C]TMSX was displaceable by just 40% with carrier and 8-(3-chlorostyryl)caffeine (each in A_2A_-AR-saturable concentrations), which may be caused by a higher amount of unspecific binding of this tracer, as it was shown for the binding in brain regions with low A_2A_-AR expression [29,30]. Additionally, it was shown that 60 min post-injection [^11^C]TMSX was very stable in human plasma (> 90% intact radioligand), whereas in mice, only 54% in plasma and 76% in heart tissue were measured [20]. In a recent study, we found that 71% of [^18^F]FLUDA was non-metabolised in plasma samples of healthy mice 15 min post-injection [18]. Hence, further studies are needed to clarify a potential enrichment of [^18^F]FLUDA-derived metabolites in the cardiac tissue over time.

Clinical outlook: [^18^F]FLUDA PET imaging could be useful to assess the receptor occupancy in the heart by A_2A_-AR-targeting drugs. This might be useful when treating Parkinsonian patients with A_2A_ antagonists to avoid adverse side effects. Another potential application could be the non-invasive determination of an elevated A_2A_AR density in patients with fibrillation as a diagnostic marker. Hence, it would be possible to establish a cause-effect relationship between A_2A_-AR density and atrial fibrillation in a non-invasive manner in long-term follow-up. The next step will be to test the eligibility of [^18^F]FLUDA in patients and to determine the uptake of [^18^F]FLUDA in the heart.

In summary, we describe a novel radioligand to label and quantify A_2A_ receptors for diagnostic purposes in the living mammalian heart.

## 4. Materials and Methods

### 4.1. General Information

All chemicals and reagents were purchased from commercially available sources and used without further purification.

The chemical– and radiosynthesis of [^18^F]FLUDA are described elsewhere [18].

### 4.2. Animals

For the present study, female FVB/N mice (A_2A_-AR TG and WT, age: 4–6 months, weight 27 ± 2 g) were used. The generation of the transgenic FVB/N mice overexpressing the human A_2A_-AR under control of an alpha myosin heavy chain promoter in cardiac tissue (A_2A_-AR TG), were described elsewhere [21]. In the present study, female A_2A_-AR TG and wild type (WT) mice were used in the indicated number. The investigation conforms to the Guide for the Care and Use of Laboratory Animals published by the National Research Council (2011). Animals were handled and maintained according to approved protocols of the animal welfare committee of the University of Münster, Germany. All procedures performed in studies involving animals were in accordance with the ethical standards of the institution or practice at which the studies were conducted (Landesdirektion Sachsen, TVV 18/18).

### 4.3. Human Atrial Preparations

Right atrium samples were obtained from patients undergoing open-heart surgery with coronary artery bypass grafts and electrically stimulated in organ baths, as described previously [31,32]. This study complied with the Declaration of Helsinki and was approved by the local ethics committee (hm-bü04.08.2005). All patients gave informed consent.

### 4.4. Contractile Function

The contractile function of mouse left atrial preparations were performed as previously described [21,31]. Control conditions were obtained by 1 µg/mL adenosine deaminase (ADA; Roche Diagnostics Deutschland GmbH, Mannheim, Germany), 1 µM of A_1_-AR antagonist 8-cyclopentyl-1,3-dipropylxanthine (DPCPX; TOCRIS, Bio-Techne GmbH, Wiesbaden-Nordenstadt, Germany), and for the CGS 21680 (Sigma-Aldrich Chemie GmbH, Taufkirchen, Germany) antagonisation, 1 µM FLUDA instead of 1 µM ZM 241385 (TOCRIS, Bio-Techne GmbH, Wiesbaden-Nordenstadt, Germany) was used.

### 4.5. In Vitro Autoradiography

Tissues (heart, muscle, and lung) were obtained from FVB/N mice, frozen in isopentane, whereas patient samples were directly frozen in liquid nitrogen. The tissues were cut using a cryostat (MICROM HM560; Fisher Scientific GmbH; Schwerte, Germany), thaw-mounted onto microscope slides, and, after air-drying, were stored at −80 °C until use. The cryosections (20 µm) from WT and hA_2A_-AR TG-mice (*n* = 3) were thawed, dried in a stream of cold air, and preincubated for 15 min with buffer (50 mM TRIS-HCl, pH 7.4, 100 mM NaCl, 5 mM MgCl_2_, 1 mM EDTA) containing 1 µU/mL adenosine deaminase (ADA, Sigma) at room temperature. Afterwards, the sections were incubated for 90 min with ~ 0.1 MBq/mL (96–340 GBq/µmol; 0.45–0.97 nM) [^18^F]-FLUDA (total binding) or with addition of 10 µM ZM241385 (non-specific binding). The homologous competition assays were performed in the presence of FLUDA in the concertation range 10^−6^ to 10^−12^. The sections were then washed twice with ice-cold 50 mM TRIS-HCl (pH 7.4), dipped in ice-cold deionised water, dried in a stream of cold air, and exposed overnight on an imaging plate. The plates were scanned using an image plate scanner (HD-CR 35; Duerr NDT GmbH; Bietigheim-Bissingen, Germany). The digitized autoradiograms were analyzed with AIDA 2.31 software (raytest Isotopenmessgeräte GmbH; Straubenhardt, Germany). The *B*_max_ and *K*_D_ values were calculated from the homologous competition of [^18^F]FLUDA with FLUDA using the Cheng-Prusoff equation (K_D_ = IC50 − [[^18^F]FLUDA] and B_max_ = Top - Bottom (K_D_ + [[^18^F]FLUDA])/[[^18^F]FLUDA]) [33]. Inhibition curves were created with GraphPad Prism 4.1 (GraphPad Inc.; La Jolla, CA, USA).

Ex vivo autoradiography of the murine hearts was performed immediately after euthanising the animals at 15 min p.i. of the radioligand. The hearts were isolated, frozen by immersion in isopentane at −20 °C, cryosectioned (16 µm; MICROM HM560), and the dried sections were exposed to a phosphor imaging plate for 120 min and processed as described above.

### 4.6. Small-Animal PET/MR Experiments

The animals were initially anaesthetised with 5% isoflurane and were positioned prone into a small-animal PET/MR (nanoScan, MEDISO, Budapest, Hungary) on a temperature-controlled bed system (37 °C) while respiration rate was continuously monitored. The anaesthesia (Anaesthesia Unit U-410, agntho’s, Lidingö, Sweden) was maintained at 2.1–1.3% isoflurane in a 60% oxygen/40% air gas mixture (Gas blender 100 series, MCQ Instruments, Rome, Italy) with 250 mL/min airflow. Prior to the 60 min PET scan, a scout image MR sequence was performed to outline the animal dimensions. Animals (A_2A_-TG and WT) received an i.v. injection of [^18^F]FLUDA (3.5 to 11.3 MBq; 1.5 to 11.3 fmol/g bodyweight). Control animals (WT: *n* = 6; A_2A_-TG: *n* = 8) received a vehicle solution containing DMSO/Kolliphor/NaCl, 1:2:7 (v/v/v) 10 min prior to [^18^F]FLUDA i.v. injection. For the determination of the A_2A_AR-specific uptake, animals received an i.v. injection of 2.5 mg/kg bodyweight tozadenant (WT and A_2A_-TG: *n* = 4) or 1mg/kg istradyfelline (WT and A_2A_-TG: *n* = 3) 10 min prior the radiotracer administration. The data were collected in list mode (11 × 10; 1 × 20; 5 × 30; 1 × 45; 4 × 60; 1 × 180; 6 × 300; 2 × 900 s). Subsequently, after the PET scan, a T1-weighted whole-body MR scan (gradient echo sequence, TR = 20 ms, TE = 3.2 ms) was performed for anatomical orientation and attenuation correction at the reconstruction step (3D-OSEM, 4 iterations, 6 subsets; MR-based attenuation correction). The reconstructed images were analysed with PMOD (Version 3.802). For analysing the [^18^F]FLUDA uptake into the myocardium and the activity concentration in the left ventricle (blood compartment), VOIs were delineated in an averaged image of the first 10 min of the PET imaging after injection of [^18^F]FLUDA, and co-registered T1 images from MR. Non-parametrical analyses of achieved time-activity curves (TACs) were performed with Microsoft Excel to determine the time to peak, the TAC peak value, and the area-under-the-curve (AUC):

(1) $$AUC_{0-t\left(x\right)}=\int_{0}^{t\left(x\right)}c\left(radioactivity\right)\times dt$$

where *c* (radioactivity) is expressed as a standardised uptake value normalised to the bodyweight in g (SUV). GraphPad Prism 9 (GraphPad Inc.; La Jolla, CA, USA) was used for graphical presentation.

### 4.7. Statistical Analysis

Values are represented as mean ± standard error of the mean (SEM). Statistical analyses were performed with Microsoft Excel and GraphPad Prism (v9, San Diego, CA, USA), with *p*-values ≤ 0.05, calculated by variance analysis (ANOVA) with Bonferroni’s post-hoc test or Student’s *t*-test if applicable, were considered as significant.

## Figures and Tables

**Table 1:** Non-compartmental analysis of [^18^F]FLUDA showing the time to peak, the time-activity curve peak value (TAC peak value), the accumulated uptake from 0 to 60 min (AUC_0–60_), and from the initial 10 min (AUC_1–10_) p.i. in investigated tissues of the WT (*n* = 5) vs. A_2A_-AR TG (*n* = 6).

Tissue	Time to Peak (min)			TAC Peak Value (SUV)			AUC_0–60_ (SUV·min)			AUC_1–10_ (SUV·min)		
	WT	TG	*p*-Value	WT	TG	*p*-Value	WT	TG	*p*-Value	WT	TG	*p*-Value
Myocardium	0.6 ± 0.1	0.6 ± 0.0	*0.105*	6.3 ± 0.6	8.1 ± 0.7	*0.0437*	19.6 ± 2.0	35.0 ± 3.1	*0.001*	8.4 ± 0.9	19.3 ± 2.2	*< 0.001*
Blood	0.7 ± 0.0	0.5 ± 0.0	*0.011*	6.9 ± 0.9	7.7 ± 0.7	*0.242*	20.4 ± 2.4	29.0 ± 1.8	*0.008*	8.6 ± 1.2	13.9 ± 1.1	*0.004*
Muscle	3.3 ± 1.0	4.0 ± 0.9	*0.303*	0.5 ± 0.1	0.5 ± 0.1	*0.327*	9.6 ± 1.4	12.1 ± 1.9	*0.153*	3.8 ± 0.5	4.7 ± 0.8	*0.175*
Lung	0.5 ± 0.1	0.5 ± 0.0	*0.500*	4.2 ± 0.4	4.7 ± 0.4	*0.243*	5.4 ± 0.5	17.8 ± 3.7	*0.135*	7.5 ± 0.7	7.3 ± 1.5	*0.128*
Liver	3.5 ± 0.5	5.1 ± 1.3	*0.123*	6.7 ± 1.1	6.1 ± 0.5	*0.313*	126 ± 17	143 ± 9	*0.200*	74.4 ± 9.3	61.5 ± 4.4	*0.119*

mean ± SEM; *p*-value—Student‘s *t*-Test.

**Table 2:** Impact of pre-treatment with istradefylline (*n* = 3) on kinetic parameters in presented tissues of WT (*n* = 6) and A_2A_-AR TG shown in % of control.

A_2A_-AR Group	Tissue	Time to Peak		TAC Peak Value		AUC_0–60_		AUC_1–10_	
		Istradyfelline	*p*-Value	Istradyfelline	*p*-Value	Istradyfelline	*p*-Value	Istradyfelline	*p*-Value
WT	Myocardium	87 ± 9	*0.175*	130 ± 18	*0.082*	116 ± 4	*0.166*	87 ± 9	*0.175*
	Blood	75 ± 0	*0.017*	125 ± 11	*0.125*	117 ± 6	*0.179*	106 ± 6	*0.381*
	Muscle	44 ± 21	*0.138*	180 ± 24	*0.027*	119 ± 17	*0.229*	142 ± 19	*0.061*
	Lung	84 ± 11	*0.223*	109 ± 31	*0.364*	131 ± 32	*0.130*	133 ± 38	*0.146*
	Liver	100 ± 25	*0.500*	100 ± 11	*0.499*	83 ± 3	*0.210*	94 ± 5	*0.410*
TG	Myocardium	120 ± 17	*0.107*	91 ± 15	*0.302*	78 ± 8	*0.083*	61 ± 9	*0.032*
	Blood	105 ± 11	*0.313*	98 ± 9	*0.442*	100 ± 11	*0.500*	90 ± 9	*0.222*
	Muscle	58 ± 13	*0.130*	138 ± 50	*0.211*	84 ± 14	*0.268*	99 ± 21	*0.483*
	Lung	116 ± 11	*0.090*	95 ± 6	*0.380*	102 ± 2	*0.470*	92 ± 5	*0.401*
	Liver	54 ± 12	*0.124*	117 ± 3	*0.090*	95 ± 5	*0.315*	102 ± 0	*0.431*

mean ± SEM; *p*-value—Student‘s *t*-test.

**Table 3:** Tissue uptake over time of [^18^F]FLUDA normalised to the left ventricle or the muscle in the initial phase from 1 to 10 min p.i. in the WT (*n* = 5) vs. A_2A_-AR TG group (*n* = 6).

Treatment	Tissue	AUC_1–10_ Ratio (Tissue-to-Left Ventricle)			AUC_1–10_ Ratio (Tissue-to-Muscle)		
		WT	A_2A_-AR TG	*p*-Value	WT	A_2A_-AR TG	*p*-Value
Vehicle	Myocardium	1.0 ± 0.0	1.4 ± 0.1	*0.001*	2.4 ± 0.5	4.8 ± 1.2	*0.028*
	Lung	0.7 ± 0.1	0.5 ± 0.1	*0.081*	1.5 ± 0.2	1.7 ± 0.2	0.297
	Liver	7.8 ± 1.0	6.0 ± 1.6	*0.190*	20.6 ± 2.8	16.5 ± 4.4	*0.223*
	Myocardium	1.0 ± 0.0	0.9 ± 0.1	*0.570*	1.8 ± 0.4	3.0 ± 1.1	*0.334*
Istradefylline	Lung	0.8 ± 0.2	0.6 ± 0.1	*0.400*	1.3 ± 0.2	1.6 ± 0.3	*0.483*
	Liver	6.5 ± 0.4	5.2 ± 0.8	*0.193*	11.5 ± 3.2	14.6 ± 2.7	*0.390*

mean ± SEM; *p*-value—Student‘s *t*-test.

## Data Availability

The data that support the findings of this study are available from the corresponding author upon reasonable request.

## Supplementary Materials

The following are available online at https://www.mdpi.com/article/10.3390/ijms23031025/s1.
