# Supplementary material for: Non-Invasive Assessment of Locally Overexpressed Human Adenosine 2A Receptors in the Heart of Transgenic Mice
Source: Int J Mol Sci. 2022 Jan 18;23(3):1025. doi: 10.3390/ijms23031025 (PMC8835051; doi:10.3390/ijms23031025)
Supplement: Supplementary file 1 [file ijms-23-01025-s001.zip › ijms-1542565 supplementary.pdf]

International Journal of *Molecular Sciences*

MDPI

Supplementary Information

Non-invasive assessment of a local overexpressed human Adenosine 2A Receptor with [^{18}F]FLUDA in the heart of a transgenic mouse model by PET

Daniel Gündel [1*], Thu Hang Lai [1,2], Sladjana Dukic-Stefanovic [1], Rodrigo Teodoro [1], Winnie Deuther-Conrad [1], Magali Toussaint [1], Klaus Kopka [1,7], Rareş-Petru Moldovan [1], Peter Boknik [3], Britt Hofmann [4], Ulrich Gergs [5], Joachim Neumann [5,#] and Peter Brust [1,6,#]

[1] Helmholtz-Zentrum Dresden-Rossendorf, Institute of Radiopharmaceutical Cancer Research, Department of Neuroradiopharmaceuticals, Research Site Leipzig, 04813 Leipzig, Germany; d.guendel@hzdr.de (D.G.), t.lai@hzdr.de (T.H.L.), s.dukic-stefanovic@hzdr.de (S.D.-S.), r.teodoro@hzdr.de (R.T.), w.deuther-conrad@hzdr.de (W.D.-C.), m.toussaint@hzdr.de (M.T.), k.kopka@hzdr.de (K.K.); r.moldovan@hzdr.de (R.P.M.); p.brust@hzdr.de (P.B.)

[2] Department of Research and Development, ROTOP Pharmaka Ltd., Dresden, Germany; t.lai@hzdr.de (T.H.L.)

[3] University of Muenster, Institute for Pharmacology and Toxicology, Muenster, Germany; boknik@uni-muenster.de (P.Bo.)

[4] Martin Luther University of Halle-Wittenberg, Medical Faculty, Cardiac Surgery, Halle, Germany; britt.hofmann@uk-halle.de (B.H.)

[5] Martin Luther University of Halle-Wittenberg, Institute for Pharmacology and Toxicology, Halle, Germany; Ulrich.gergs@medizin.uni-halle.de (U.G.), joachim.neumann@medizin.uni-halle.de (J.N.)

[6] The Lübeck Institute of Experimental Dermatology, University Medical Center Schleswig-Holstein, 23562 Lübeck, Germany; p.brust@hzdr.de (P.B.)

[7] Technical University Dresden, School of Science, Faculty of Chemistry and Food Chemistry, Dresden, Germany (K.K.)

* Correspondence: d.guendel@hzdr.de; Tel.: +49 341 234179 4615

# **authors contributed equally**

Citation: Gündel, D.; Lai, T.H.; Dukic-Stefanovic, S.; Teodoro, R.; Deuther-Conrad, W.; Toussaint, M.; Kopka, K.; Moldovan, R.-P.; Boknik, P.; Hofmann, B.; et al. Non-Invasive Assessment of Locally Overexpressed Human Adenosine 2A Receptors in the Heart of Transgenic Mice. *Int. J. Mol. Sci.* **2022**, *23*, 1025. https://doi.org/10.3390/ijms23031025

Academic Editor: Katia Varani

Received: 21 December 2021
Accepted: 14 January 2022
Published: 18 January 2022

Publisher's Note: MDPI stays neutral with regard to jurisdictional claims in published maps and institutional affiliations.

Copyright: © 2022 by the authors. Submitted for possible open access publication under the terms and conditions of the Creative Commons Attribution (CC BY) license (https://creativecommons.org/licenses/by/4.0/).

Table of content:

Figure S1.: *In vitro* binding study of [^{18}F]FLUDA to control tissue cryosections (muscle and lung).

Figure S2.: Dynamic PET imaging analysis of the [^{18}F]FLUDA in the blood compartment (Left ventricle) and the radiotracer uptake of the myocardium in WT (n = 6) and A_{2A}-AR TG (n = 6) mice.

Figure S3: *Ex vivo* autoradiographic analysis of [^{18}F]FLUDA accumulation in heart.

Figure S4: Dynamic PET imaging analysis of the [^{18}F]FLUDA in different tissues.

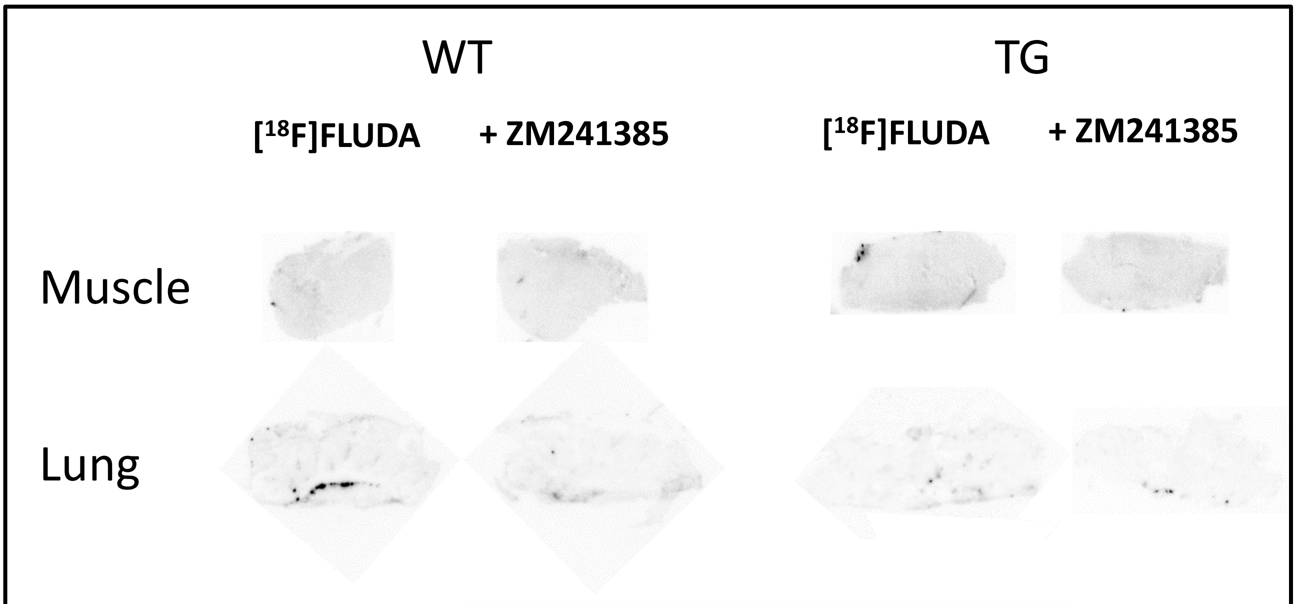

Specific binding (%)		
tissue	WT	A_{2A}AR TG
Muscle	8.2 ± 3.6	9.7 ± 7.2
lung	33.4 ± 8.9	21.3 ± 0.8

Figure S1. *In vitro* binding study of [^{18}F]FLUDA to control tissue cryosections (muscle and lung) with and without 10 µM ZM241385 (each n = 2) to determine the specific binding of the radioligand.

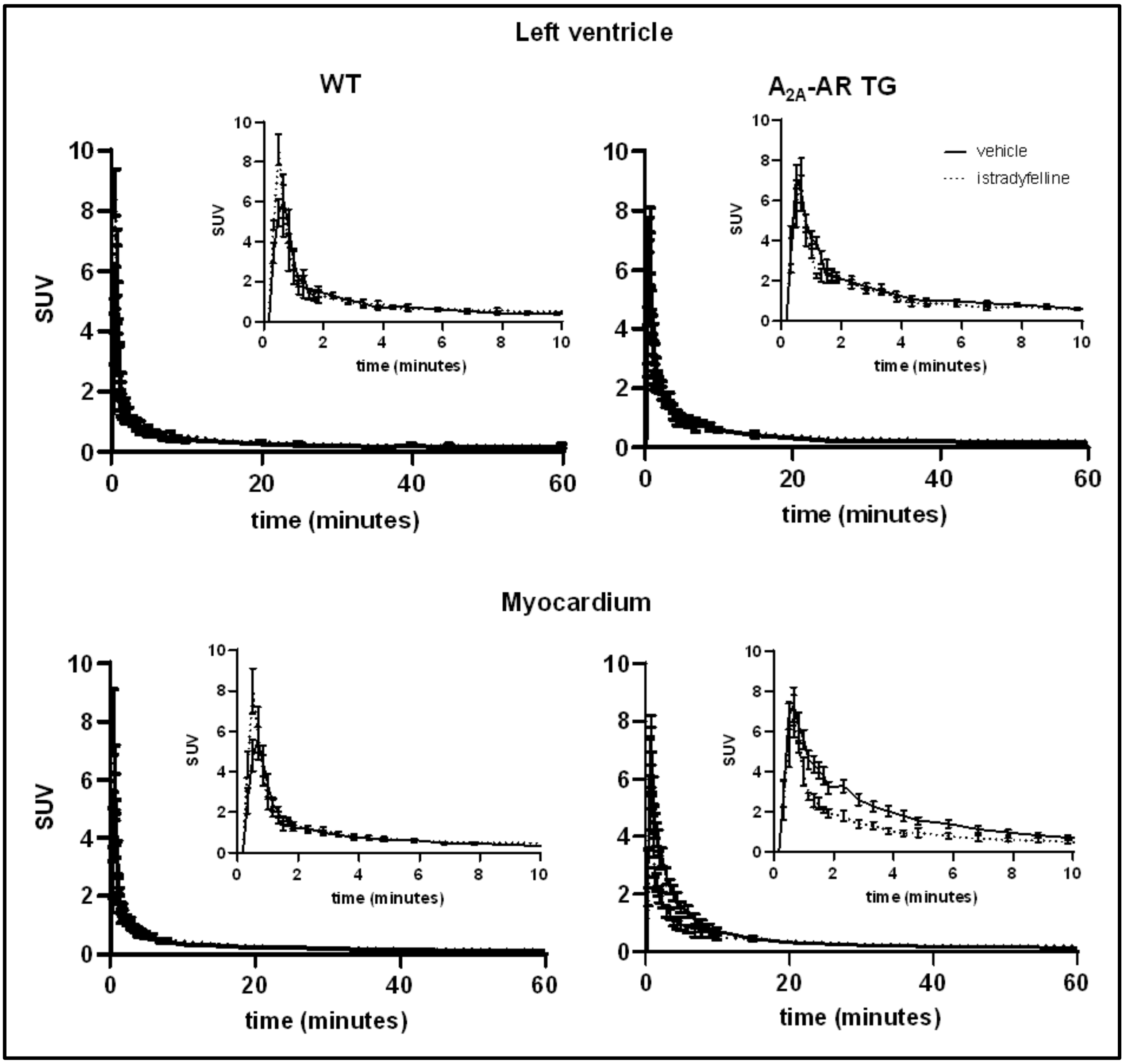

Figure S2. Dynamic PET imaging analysis of the [^{18}F]FLUDA concentration in the blood compartment (Left ventricle) and the radiotracer uptake of the myocardium in WT (n = 6) and A_{2A}-AR TG (n = 6) mice. The time activity curves after radiotracer administration are represented in mean activity concentrations as standardized uptake values (SUV_{mean}±SEM) in the delineated volume of interests of indicated tissue regions. Mice were pre-treated with vehicle or 1 mg/kg bodyweight istradefylline 10 minutes prior radiotracer application.

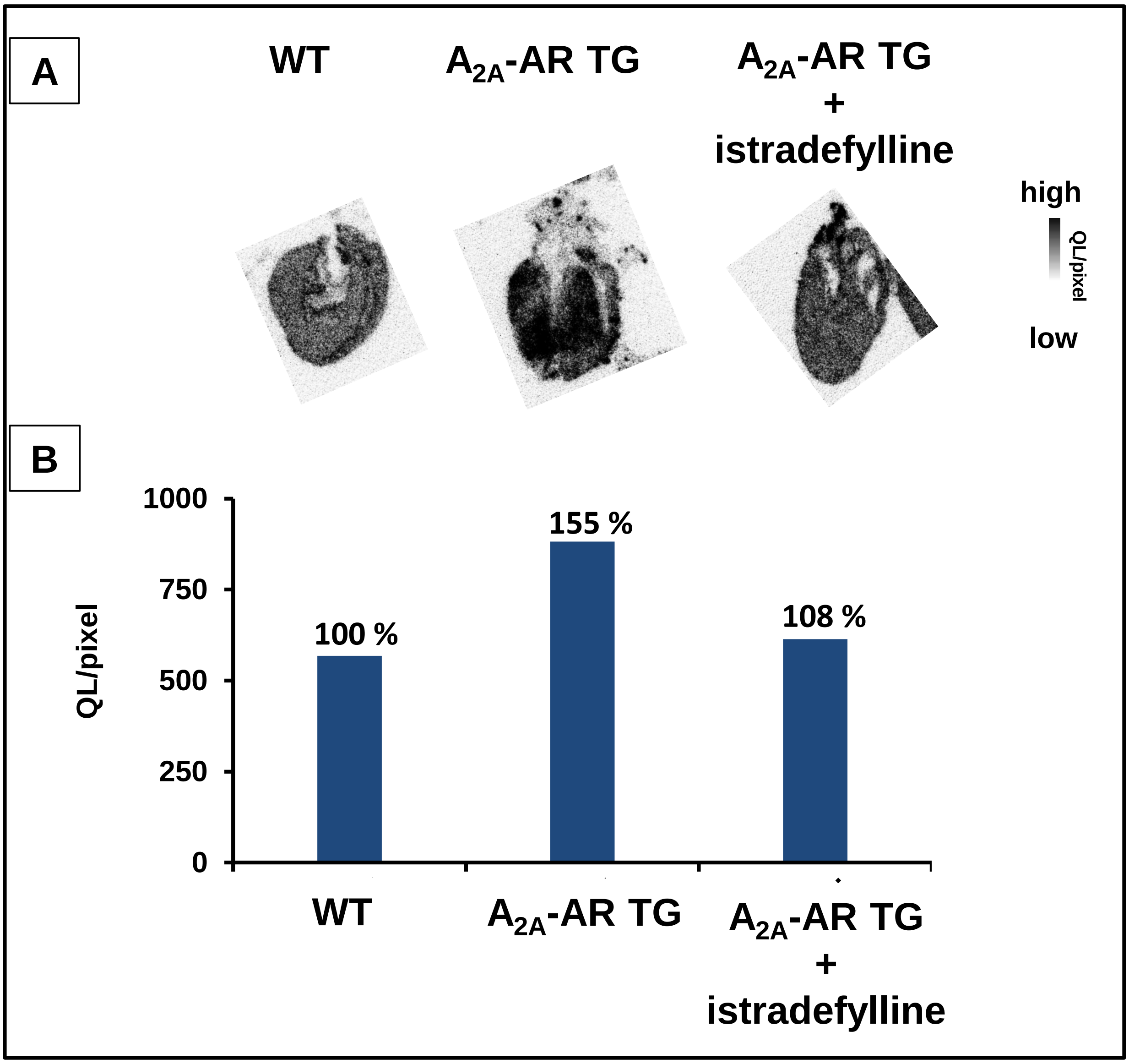

Figure S3. Exemplary *ex vivo* autoradiographic images (A) and the according analysis (B) of [^{18}F]FLUDA accumulation in heart at 15 min p.i. of the radioligand in cryosections of murine heart (16 µm) of WT and A_{2A}-AR TG (with and without preadministration of istradefylline, n=1).

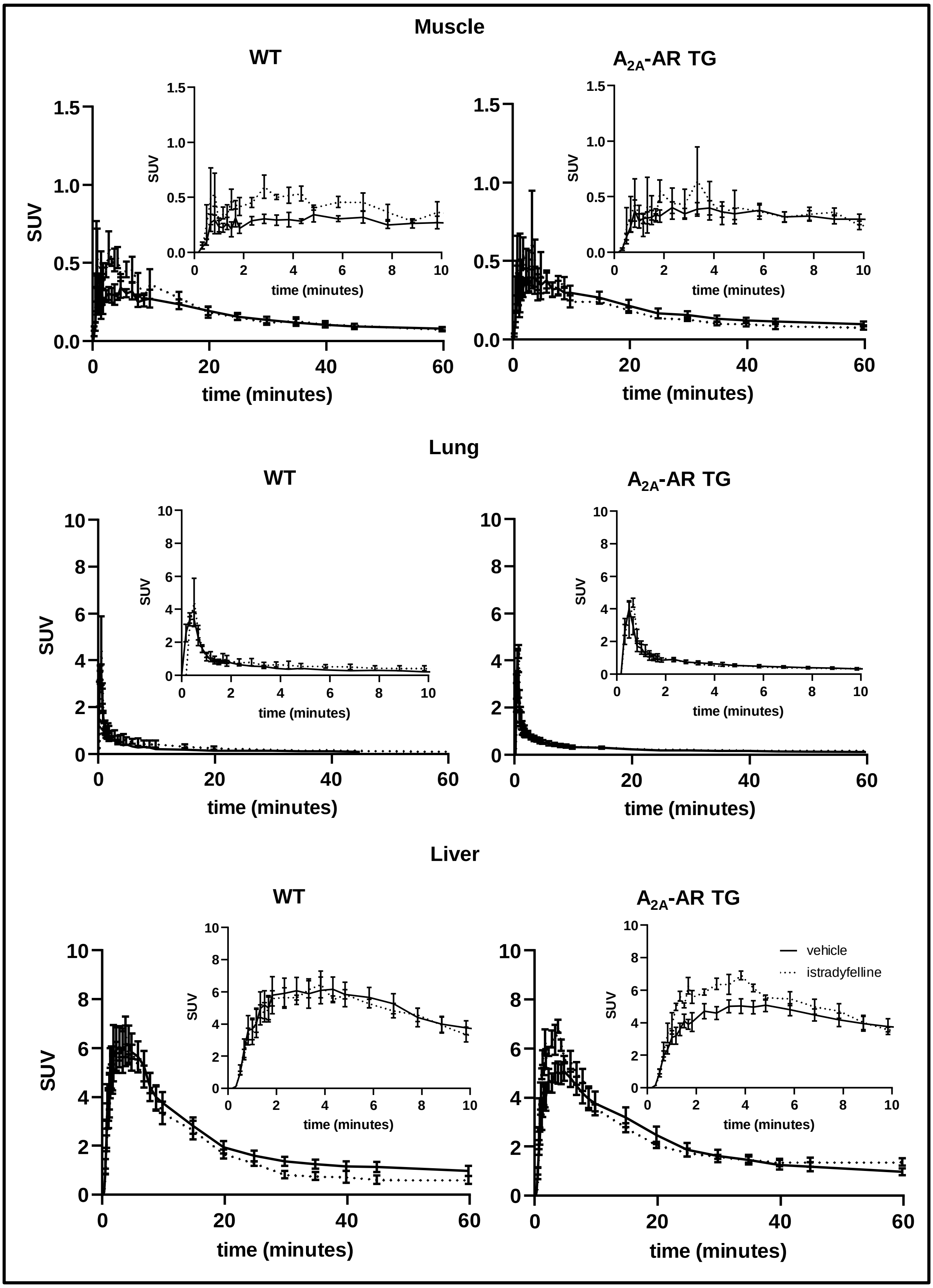

Figure S4. Dynamic PET imaging analysis of the [^{18}F]FLUDA concentration in the blood and of the radiotracer uptake of different tisssues in WT (n = 6) and A_{2A}-AR TG (n = 6) mice. The time activity curves after radiotracer administration are represented in mean activity concentrations as standardized uptake values (SUV_{mean}±SEM) in the delineated volume of interests of indicated tissue regions. Mice were pre-treated with vehicle or 1 mg/kg bodyweight istradefylline 10 minutes prior radiotracer application.

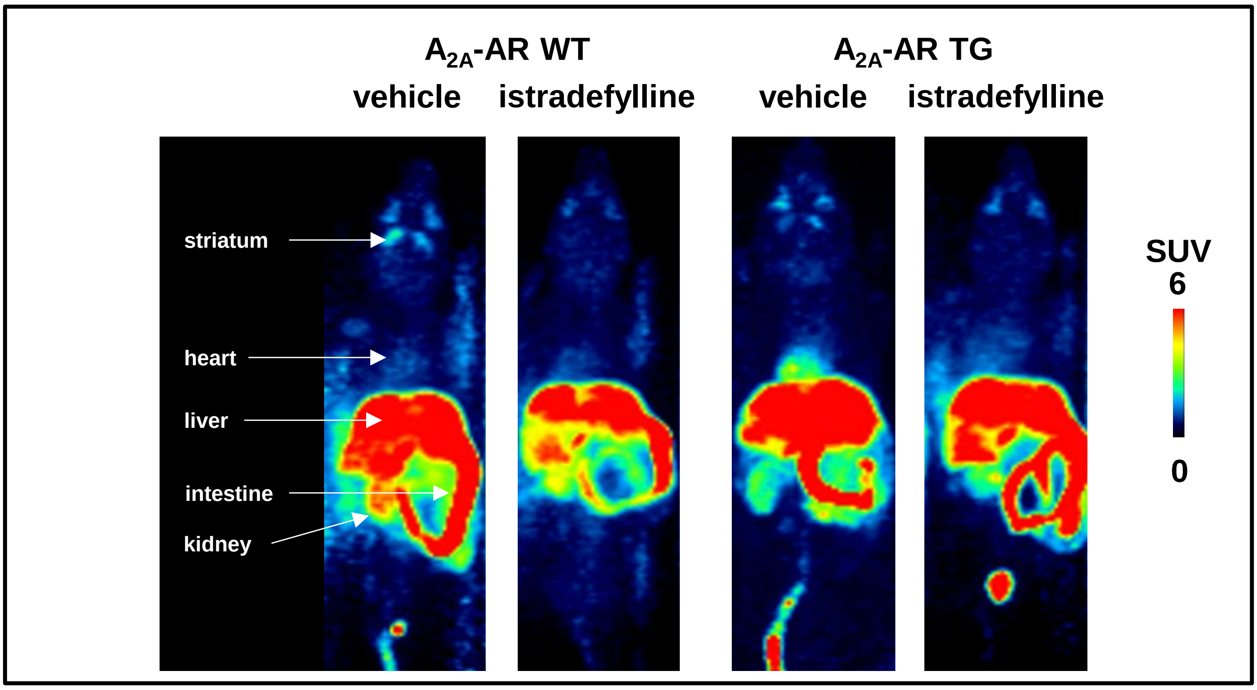

Figure S5. Representative averaged whole body maximum intensity projections (MIPs) from 1 to 10 min p.i. of the PET acquisition of [^{18}F]FLUDA in WT and A_{2A}-AR TG with or without preadministration of istradefylline.
